# The Nutrient Balance Concept: A New Quality Metric for Composite Meals and Diets

**DOI:** 10.1371/journal.pone.0130491

**Published:** 2015-07-15

**Authors:** Edward B Fern, Heribert Watzke, Denis V. Barclay, Anne Roulin, Adam Drewnowski

**Affiliations:** 1 Nestec SA, International Headquarters, Avenue Nestlé 55, Vevey, Switzerland; 2 Center for Public Health Nutrition, University of Washington, Seattle, Washington, United States of America; Indiana University, UNITED STATES

## Abstract

**Background:**

Combinations of foods that provide suitable levels of nutrients and energy are required for optimum health. Currently, however, it is difficult to define numerically what are ‘suitable levels’.

**Objective:**

To develop new metrics based on energy considerations—the Nutrient Balance Concept (NBC)—for assessing overall nutrition quality when combining foods and meals.

**Method:**

The NBC was developed using the USDA Food Composition Database (Release 27) and illustrated with their MyPlate 7-day sample menus for a 2000 calorie food pattern. The NBC concept is centered on three specific metrics for a given food, meal or diet—a Qualifying Index (QI), a Disqualifying Index (DI) and a Nutrient Balance (NB). The QI and DI were determined, respectively, from the content of 27 essential nutrients and 6 nutrients associated with negative health outcomes. The third metric, the Nutrient Balance (NB), was derived from the Qualifying Index (QI) and provided key information on the relative content of qualifying nutrients in the food. Because the Qualifying and Disqualifying Indices (QI and DI) were standardized to energy content, both become constants for a given food/meal/diet and a particular consumer age group, making it possible to develop algorithms for predicting nutrition quality when combining different foods.

**Results:**

Combining different foods into composite meals and daily diets led to improved nutrition quality as seen by QI values closer to unity (indicating nutrient density was better equilibrated with energy density), DI values below 1.0 (denoting an acceptable level of consumption of disqualifying nutrients) and increased NB values (signifying complementarity of foods and better provision of qualifying nutrients).

**Conclusion:**

The Nutrient Balance Concept (NBC) represents a new approach to nutrient profiling and the first step in the progression from the nutrient evaluation of individual foods to that of multiple foods in the context of meals and total diets.

## Introduction

Nutrient profiling is the science of ranking or classifying individual foods based on their nutrient composition [1–3]. The goal of most nutrient profiling systems is to distinguish foods that are energy dense from those that are nutrient rich [2,4]. Typically, nutrient profile models assess a food’s nutrient content per 100g, per 100 kcal or per serving. Each food is then assigned a single composite score that best reflects its overall nutritional value [1,2]. Nutrient profiling models have a variety of applications, ranging from regulatory uses [5] to the nutritional screening of product portfolios by the food industry [6] and have been variously validated with respect to expert opinion [7–9] or to independently-obtained measures of a healthy diet [10–13].

By focusing almost exclusively on individual foods, the work on nutrient profiling has yielded some paradoxes. On one hand, some low-ranked foods containing specific nutrients (e.g. monounsaturated fats) considered by many to be detrimental to health if consumed in excess are, in reality, indispensable to good health. On the other hand, some of the highly-ranked nutrient-rich foods do not contain sufficient calories to meet energy requirements [3]. Based on population food consumption patterns, different foods may deliver variable amounts of key nutrients to the total diet [14]. The relative amounts of these nutrients in culturally appropriate food consumption patterns needs to be taken into account in the new generation of nutrient profiling models [9,15,16].

Nutrient profile models have been based on qualifying nutrients only, disqualifying nutrients only, or on some combination of both [1,2,15]. Whereas qualifying nutrients have included various mixtures of protein, fiber and a variety of vitamins and minerals, disqualifying nutrients have always been those whose excessive consumption are linked to negative health concerns, as for example saturated fat, sugar, and sodium. The selection of key nutrients for inclusion in such models has mainly followed regulatory frameworks and dietary guidance [1,17] and as a result has focused primarily on a few main ones of topical importance.

Also missing from most nutrient profiling models are broader considerations of how nearly all foods can play a role in improving nutrition quality (also referred to as ‘balance’) in meals and diets. The term ‘nutrition quality’ in the context of this publication refers to the optimal interrelationship in a food between the content of qualifying (essential) and disqualifying (public health concern) nutrients relative both to their dietary recommendations and the energy content of the food.

The purpose of the current study was to examine some new approaches to nutrient profiling to see if any could describe nutrient density, energy density and the profile of qualifying nutrient in numerical terms that could be applied in precisely the same way to the whole food spectrum. Such an approach could serve to shift the current focus from judging individual foods to quantifying the added nutrition value of combining them in meals and total diets.

## Materials and Methods

The basis of the NBC was a comparison of nutrient levels that are known to be essential or important for maintaining health (qualifying nutrients) with those that are widely regarded as being detrimental to it when consumed in excess (disqualifying nutrients). The outcome was two quantitative indices–the Qualifying Index *(*QI) and the Disqualifying Index (DI)–both of which were standardized to the energy content of the food under consideration to make them independent of portion size and, as a consequence, more strictly comparable.

In addition a third metric, that of Nutrient Balance (NB), was included to indicate the capacity of a food, meal or diet to meet all the daily dietary requirements of qualifying nutrients.

### Nutrient Composition Databases and Analysis of USDA 7-Day MyPlate Sample Menus

The major nutrient composition database used for developing the Nutrient Balance Concept was the United States Department of Agriculture’s National Nutrient Database, Standard Reference, Release 27; 2014 [18]. Initial development of the concept, however, was also based on the Danish Food Composition Databank (Version 7, 2009) [19].

To illustrate the different aspects of the NBC, we have analyzed the nutrient content of meals and snacks as close as possible to those given in the USDA 7-day MyPlate sample menus [20] using the USDA Nutrient Database [18]. MyPlate 7-day sample menus are intended as a means to motivate health eating patterns by providing examples of varied daily menus based on many different food items that, over time, provide the recommended amounts of key nutrients [20].

### Qualifying (QI) and Disqualifying (DI) Indices

The current analysis included 27 qualifying nutrients that are accepted as essential, or at least very important, for maintaining health and for which DRIs have been published by the Institute of Medicine, National Academy of Sciences [21]. They were also included because nutrient compositional data existed for them in the USDA Database [18]. Known essential nutrients for which there was either no, or very limited, information on their content in foods in the USDA Database (biotin, iodine, chromium, molybdenum and fluoride) were excluded from the list of qualifying nutrients.

The Qualifying Index (QI) was defined, for the present purpose, as the ratio of each nutrient contained in 2000 kcal of a given food relative to its Dietary Reference Intake (DRI) value. Wherever possible the Recommended Dietary Allowance (RDA) for a nutrient was used but if an RDA did not exist then the Adequate Intake (AI) was used instead.

Six disqualifying nutrients were incorporated in the analysis, all chosen because of their generally recognized adverse effects on health when consumed in excess in the diet. The Disqualifying Index (DI) was defined in a very similar way to that of the Qualifying Index (QI)–namely, as the ratio of the amount of nutrient contained in 2000 kcal of a food, relative to the daily Maximal Reference Values (MRV) for that nutrient. The specific disqualifying nutrients and the MRVs used were: total fats (<35% of energy); saturated fats (<10% of dietary energy), cholesterol (<300 mg), trans fats (<1%) and sodium (<2300 mg), all as specified in the Dietary Guidelines for Americans [22], as well as total sugar (<25% of dietary energy, based on recommendations from the Institute of Medicine, USA [23]).

Alcohol (ethyl) was not included as a disqualifying nutrient in the current analyses because it was not present in individual foods other than alcoholic beverages and because the MyPlate sample menus, used to illustrate the applicability of the NBC, omit alcohol altogether. Nonetheless, alcohol can be a significant component of dietary energy intake in several regions of the world and, consequently, should be included when assessing total diets in such areas.

A list of qualifying and of disqualifying nutrients are shown, respectively, in Tables 1 and 2, using fat-free milk containing 0.2% milk fat with added vitamins A and D as an example. Fat-free milk was the most frequently occurring food item in the MyPlate 7-day menus.

**Table 1:** Derivation of ‘Qualifying Index’ (QI) and ‘Nutrient Balance’ (NB) for non-fat milk with added vitamins A & D ^1^.

Energy or Qualifying Nutrient	Unit	DRI Female 19–50 years ^2^	Amount in 2000 kcal	Qualifying Index of each nutrient (qi) = proportion of DRI in 2000 kcal ^3^	Value of ‘qi’ taken to calculate Nutrient Balance (NB) ^4^
Energy	kcal	2000	2000	(1.00)	
*Vitamins*					
Folate	μg	400	289.2	0.72	0.72
Niacin ^5^	mg	14	5.54	0.40	0.40
Pantothenic Acid	mg	5	21.08	4.22	1.00
Riboflavin	mg	1.1	10.75	9.77	1.00
Thiamin	mg	1.2	2.65	2.21	1.00
Vitamin A ^6^	μg	700	3590.4	5.13	1.00
Vitamin B12	μg	2.4	29.40	12.25	1.00
Vitamin B6	mg	1.3	2.19	1.69	1.00
Vitamin C	mg	75	0.00	0.00	0.00
Vitamin D ^7^	μg	7.5	69.88	9.32	1.00
Vitamin E	mg	15	0.48	0.03	0.03
Vitamin K	μg	90	0.00	0.00	0.00
*Minerals and Trace Elements*					
Calcium	mg	1000	7204.8	7.21	1.00
Copper	mg	0.9	0.77	0.86	0.86
Iron	mg	18	1.69	0.09	0.09
Magnesium	mg	320	650.6	2.03	1.00
Manganese	mg	1.8	0.17	0.09	0.09
Phosphorus	mg	700	5951.8	8.50	1.00
Potassium	mg	4700	9204.8	1.96	1.00
Selenium	μg	55	183.1	3.33	1.00
Zinc	mg	8	24.82	3.10	1.00
*Other Qualifying Nutrients*					
α-Linolenic Acid	g	1.1	0.05	0.05	0.05
Linoleic Acid	g	12	0.12	0.01	0.01
Choline	mg	425	920.5	2.17	1.00
Dietary Fiber	g	25	0.00	0.00	0.00
Protein	g	46	199.0	4.33	1.00
Water	g	2700	5362.9	1.99	1.00
**Qualifying Index (QI) of food (= arithmetic mean of ‘qi *‘* values of each qualifying nutrient)**				**3.02**	
**Nutrient Balance (NB) of food (= arithmetic mean of truncated and non-truncated ‘qi ‘ values for each qualifying nutrient, expressed as a percentage of total number of qualifying nutrients considered)**					**67.6%**

1 USDA National Nutrient Database for Standard Reference (Release 27; 2014).

2 DRI—Dietary Reference Intakes (Food & Nutrition Board, Institute of Medicine, National Academy of Sciences, USA) for women (19–50 years of age) taken as representative for a general population.

3 Qualifying Index of a nutrient (qi) = ratio of DRI of nutrient to DRI of energy in 2000 kcal.

4 Nutrient Balance of food (NB) = provision of each qualifying nutrient relative to DRI (values of qi greater than 1.0 have been truncated to 1.0—see text).

5 Actual Niacin content (not niacin equivalents).

6 Retinol Activity Equivalents (RAE).

7 Vitamin D content taken as Vitamin D2 + D3.

**Table 2:** Derivation of ‘Disqualifying Index’ (*DI*) for non-fat milk (with added vitamins A & D ^1^).

Energy or Disqualifying Nutrient	Unit	MRV for adults ^2^ (DRI for Energy)	Amount in 2000 kcal	Disqualifying Index (di) for each nutrient (= proportion of MRV in 2000 kcal ^3^)
Energy	kcal	2000	2000	
Total Fats	g	78	4.82	0.06
Saturated Fats	g	22	3.30	0.15
*Trans* Fatty Acids	g	2.22	0.00	0.00
Cholesterol	mg	300	120.5	0.40
Total Sugars	g	125	300.5	2.40
Sodium	mg	2400	2482	1.03
	**Disqualifying Index (*DI*) of food (= arithmetic mean of *di* values)**			**0.67**

1 USDA National Nutrient Database for Standard Reference (Release 27; 2014).

2 MRV—Maximum Reference Values per day (taken from various sources–see text).

3 Disqualifying Index of nutrients (di) = ratio of MRV of nutrient to DRI of energy in 2000 kcal.

### Derivations of QI and DI Indices

Both the Qualifying (QI) and Disqualifying (DI) Index were expressed relative to a daily energy intake of 2000 kcal, as arithmetic means of the proportions of the amounts of each nutrient in a given food relative to the published DRI for that nutrient. The 2000 kcal/day value was chosen because it is widely accepted as the daily average amount of energy required for the general population. However, when considering specific age groups, such as children, adolescents and adult males, their respective daily energy and nutrient requirements should be used.

Mathematically, the Qualifying Index (QI) is given by (Eq 1):`

$$QI=\frac{E_{d}}{E_{p}}\cdot \frac{\sum_{j=1}^{N_{q}}\frac{a_{q,j}}{r_{q,j}}}{N_{q}}$$

and the Disqualifying Index (DI) by (Eq 2):

$$DI=\frac{E_{d}}{E_{p}}\cdot \frac{\sum_{j=1}^{N_{d}}\frac{a_{d,j}}{r_{d,j}}}{N_{d}}$$

where:

*E*_*d*_ = daily energy needs of the population age group under consideration (kcal)

*E*_*p*_ = energy in the amount of the food or meal analyzed (kcal)

*a*_*q*,*j*_ = amount of qualifying nutrients in the food analyzed (g, mg or mcg)

*a*_*d*,*j*_ = amounts of disqualifying nutrients in the same amount of the food (g or mg)

*r*_*q*,*j*_ = DRI of qualifying nutrients (g, mg or mcg/day)

*r*_*d*,*j*_ = MRV of disqualifying nutrients (g or mg/day)

*N*_*q*_ = number of qualifying nutrients considered

*N*_*d*_ = number of disqualifying nutrients considered

The empirical approach for calculating QI and DI are shown in Tables 1 and 2.

If the QI value was above 1.0, the food was considered as nutrient dense; the greater the value, the higher the nutrient density. By contrast, foods with a QI less than 1.0 were considered as energy dense; the smaller the value, the greater the energy density.

If the DI value was greater than 1.0, a food was regarded as being compromised by disqualifying nutrients because, relative to energy content, the average level of these nutrients exceeded those specified by MRVs. DI values below 1.0 signified that a food was not compromised—for the opposite reason.

### Calculating Qualifying and Disqualifying Indices for Composite Meals and Snacks

The values for the Qualifying and Disqualifying Indices for composite meals (QI _composite_ and DI _composite_, respectively) were calculated as the sum of weighted values for the individual component foods, weighted according to their energy contribution to the total energy content of the meal (designated ‘e_i_’ in Eqs 3 and 4).

The composite Qualifying Index (QI _composite_) is represented mathematically by (Eq 3):

$$QI_{composite}=\sum_{i=1}^{k}e_{\text{i}}Q_{i}$$

and that of the composite Disqualifying Index (DI _composite_) by (Eq 4):

$$DI_{composite}=\sum_{i=1}^{k}e_{\text{i}}D_{i}$$

where:

*Qi* = Qualifying index of a component food in the combination

*QI*_*composite*_ = Qualifying Index of the combination—based on QI of each component food, weighted for its contribution to total energy

*D*_*i*_ = Disqualifying Index of a component food in the combination

*DI*_*composite*_ = Disqualifying Index of the combination—based on DI of each component food, weighted for its contribution to total energy

*e*_*i*_ = proportion of total energy contributed by each food component

*k* = number of combined foods

The way that the above calculations were made in practice is shown in Table 3, using the MyPlate breakfast meal on Day 1 as an example.

**Table 3:** Derivation of *DI* and *QI* values for a composite meal (‘My Plate’, Day 1, Breakfast ^1^)

			Disqualifying Index		Qualifying Index	
Food Item	USDA Database Code ^2^	Portion of total energy of meal	DI value of food	Weighted contribution to DI value of meal	QI value of food	Weighted contribution to QI value of meal
		*a*	*b*	*ab*	*c*	*ac*
Uncooked oatmeal (*½ cup)*	*08120*	0.309	0.126	**0.039**	1.349	**0.417**
Unsweetened orange juice (1 *cup)*	*09206*	0.224	0.526	**0.118**	2.015	**0.453**
Brown sugar (*2 teaspoons)*	*19334*	0.191	0.691	**0.132**	0.073	**0.014**
Fat-free milk (*1 cup)*	*01085*	0.167	0.675	**0.113**	3.016	**0.503**
Raisins (*2 tablespoons)*	*09298*	0.108	0.544	**0.059**	0.456	**0.049**
**Composite meal (= sum of food items)**		1.00		**0.46**		**1.44**

1 “My Table—Sample Menus for a 2000 calorie Food Pattern—Day 1 Breakfast” used as the example [20].

USDA National Nutrient Database for Standard Reference–Release 27, 2014 [18].

### The Qualifying Nutrient Balance (NB) Score

The NB score is an indicator of the extent to which a food, meal or diet can satisfy the daily requirements for all qualifying nutrients present in a sample containing 2000 kcal. If a food satisfies the daily dietary requirement for every qualifying nutrient, its NB score would be the maximum value of 100%. Conversely, a value of 0% would denote that none of the requirements for qualifying nutrients were met at all by the food. The intermediate conditions have a linear relationship, which allows direct comparisons to be made between individual foods, meals and diets.

Mathematically, NB *(*expressed as a percentage) is represented by (Eq 5):

$$NB(\%)=\frac{\sum_{i=j}^{Nq}qi_{q,j}}{N_{q}}\cdot 100$$

where:

*qi*_*q*,*j*_ = value for the Qualifying Index of an individual nutrient (both truncated and non-truncated—see text below)

*Nq* = number of qualifying nutrients considered.

The Nutrient Balance (NB) is, therefore, derived from the Quailfying Index of individual qualifying nutrients (qi) with and without truncation. As shown in Table 1, the Qualifying Index of any single nutrient that was above 1.0 (qi>1.0) was truncated to 1.0. Values of any nutrient equal to or less than 1.0 (qi <1.0) were used unaltered. The rationale for this was that once the requirement for a specific qualifying nutrient is met (qi = 1.0) any further provision of that nutrient can be considered as having no additional nutrition function. It also guarded against inflated mean values for a food containing abnormally high amounts of one or two individual nutrients.

Unlike the Quailfying (QI) or Disqualifying (DI) Index, which are both readily calculated as a weighted average for different combinations of foods (see Table 3), the Nutrient Balance (NB) required a separate re-assessment to be made from basic qi values. This is because of the considerable variation in nutrient complementarity that exists when combining different foods and food components.

## Results

### The QI, DI and NB Scores of Different Food Groups

**Fig 1:**
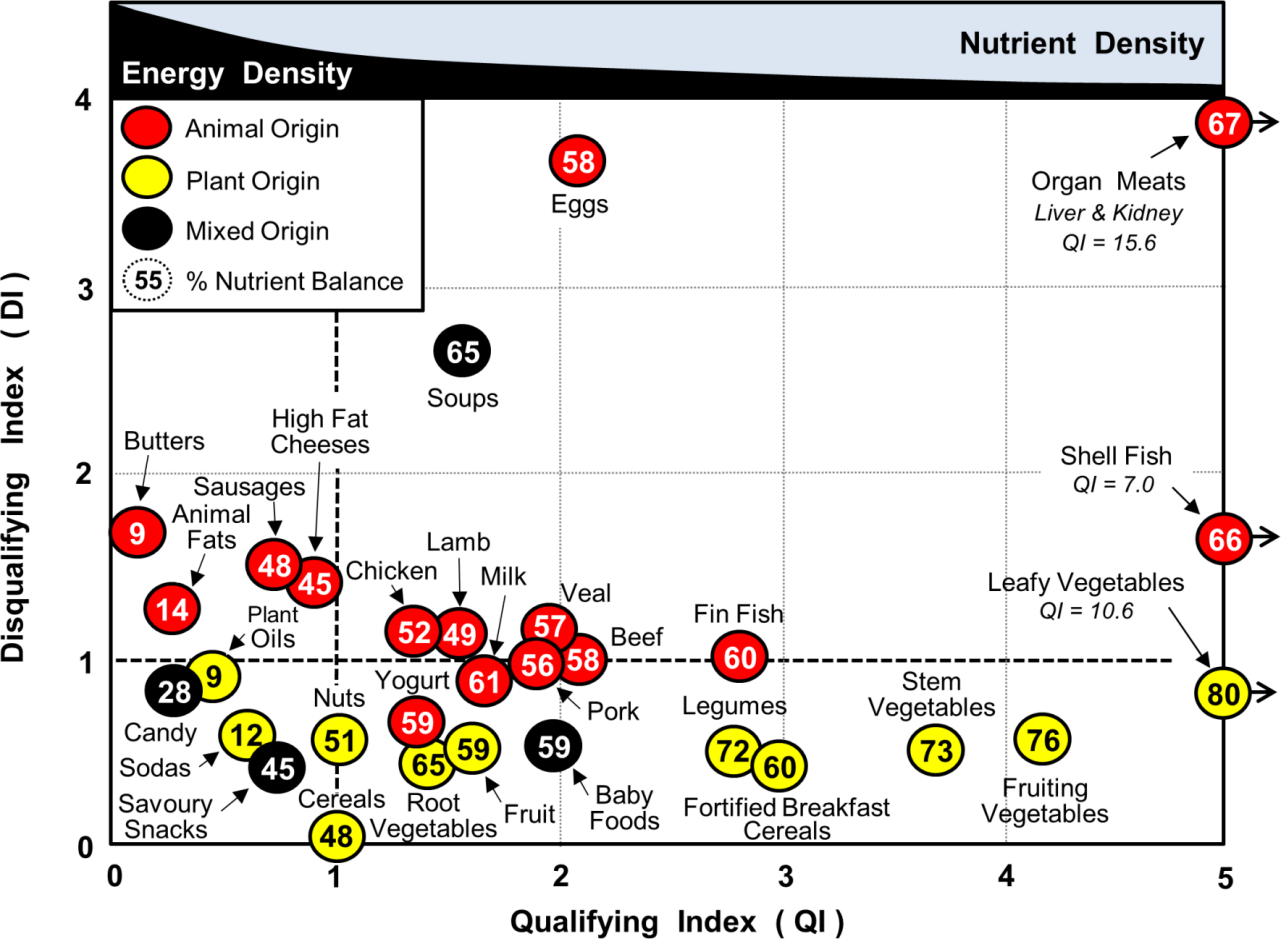
NBC Scores for typical food groups. Data shown are the Qualifying Index (QI), Disqualifying Index (DI) and % Nutrient Balance (NB, value in circle).

Fig 1 illustrates the large variation in the values of the Qualifying (QI) and Disqualifying (DI) Indices and the Nutrient Balance (NB) for different food groups. A total of 3379 foods from the USDA National Nutrient Database Standard Reference, Release 27; 2014 [18] were analyzed and assigned to aggregated food groups represented in the database. In general foods with the highest Qualifying Index (QI) paired with a low Disqualifying Index (DI) were leafy, fruiting and stem vegetables as well as legumes. Shellfish, organ meats and eggs also had high QI values but scored much higher on DI than did fruit and vegetables, mainly because of their cholesterol, sodium and/or saturated fat content. Products containing high levels of saturated animal fats, sugars and/or sodium tended to have low QI scores. The purpose of Fig 1 is simply to illustrate that although the way different food groups varied with one another reflected what is already known about nutrient densities, each food group could now be characterized numerically.

The Nutrient Balance (NB) parameter in Fig 1, however, is original and brings a completely new dimension to interpretation of nutrition quality. Broadly speaking NB values for the various food groups were directly proportional to their respective QIs but there were variations between individual food groups. The highest NB scores were obtained for leafy, fruiting and stem vegetables, as well as legumes. Medium scores were obtained for milk, meat, poultry and fish (both fin and shell), and low NB scores for oils, fats and sugars.

**Fig 2:**
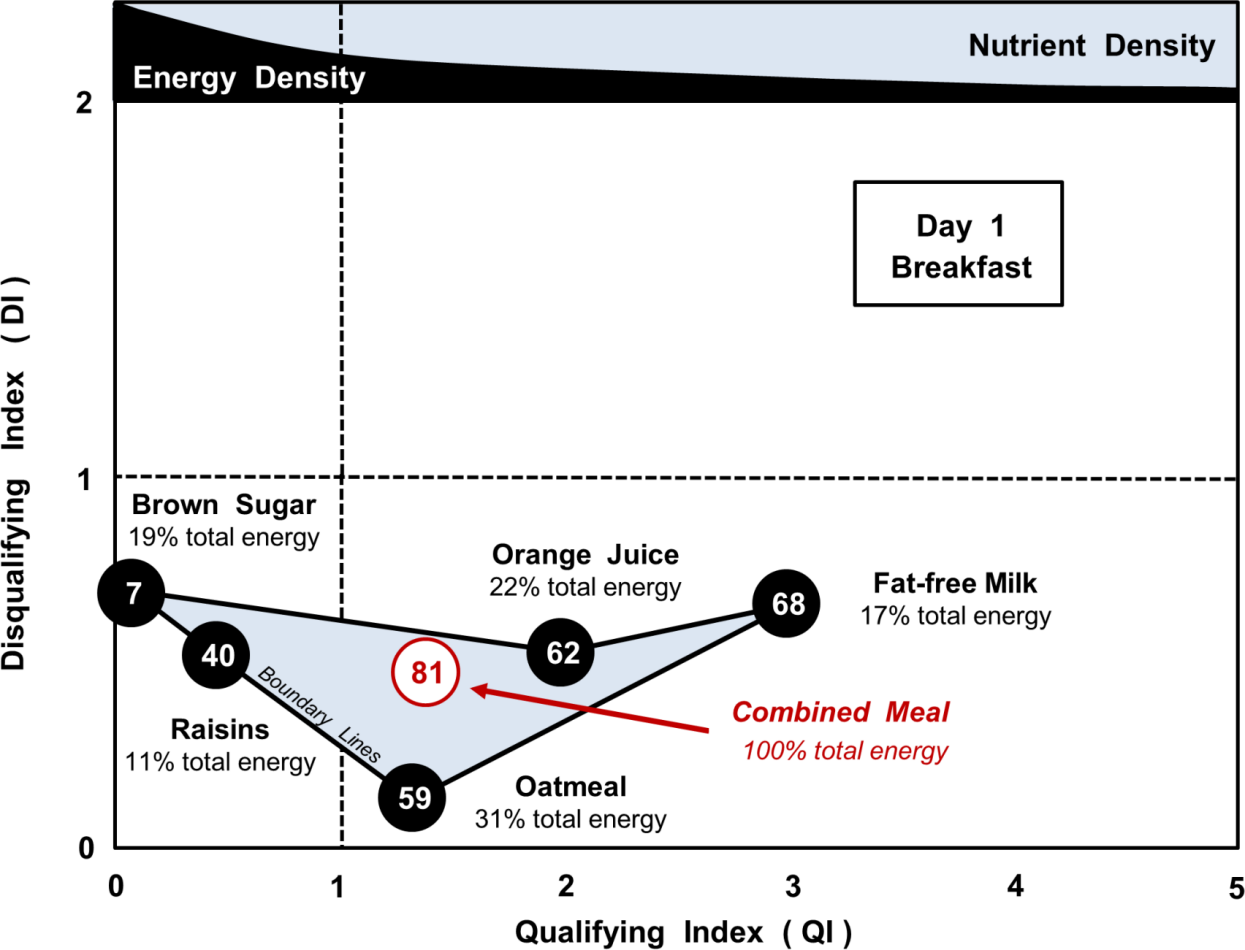
Day 1 Breakfast (MyPlate 7-day menus): NBC Scores for component foods, individually and collectively. Data shown are the Qualifying Index (QI), Disqualifying Index (DI) and % Nutrient Balance (NB, value in circle) for each meal component and the composite meal. Percentage value in each food component indicates their contribution of that food to the total energy of the meal.

Concerning individual meals, Fig 2 and Table 3 show values of the Qualifying Index (QI), Disqualifying Index (DI) and the Nutrient Balance (NB) for the five components of the MyPlate breakfast menu on Day 1, both individually and when combined in the energy proportions shown.

All foods had a DI value <1.0, indicating acceptable levels of disqualifying nutrients. Their QI scores ranged from 0.07 to 3.01, which in comparison with many of the other meals during the 7 days, was a relatively small variation.

When the food components were combined as a breakfast, the QI and DI values for the composite were given by those for each of the component foods, weighted for the energy contribution that each made to the total meal. Thus, QI and DI for the composite will be somewhere within the boundary lines joining the points representing the QI and DI coordinates for each of the five constituent foods, the exact point being governed by the energy contribution that each makes to the total for the meal.

**Fig 3:**
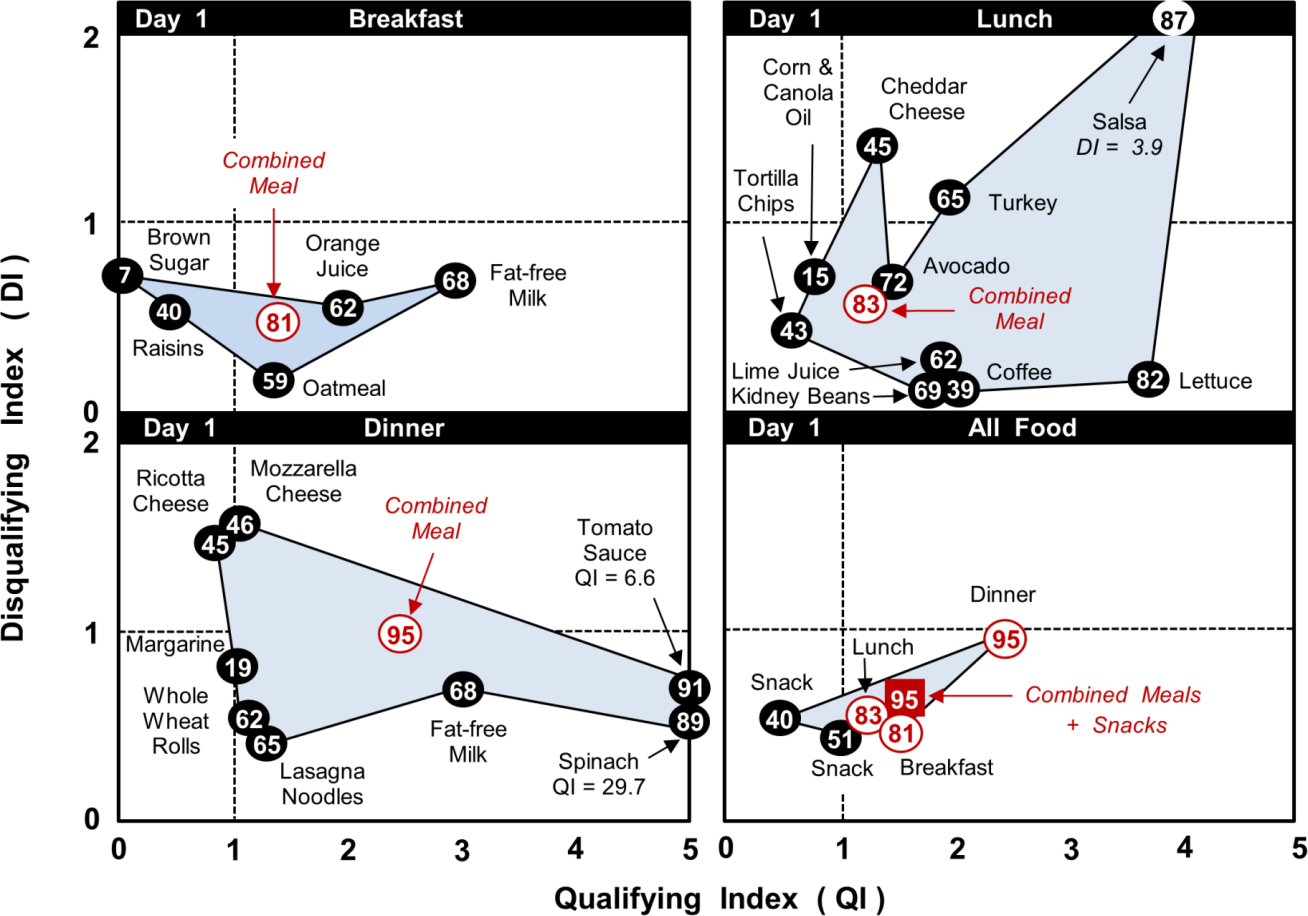
Day 1 (MyPlate 7-day menus): NBC Scores for foods, snacks and meals. Qualifying Index (QI), Disqualifying Index (DI) and % Nutrient Balance (NB, value in circle) for meal components, snacks, composite meals and the daily total food intake.

Fig 3 highlights the differences in the nutrient content of components for the breakfast, lunch and dinner on Day 1. The differences were particularly noticeable between the lunch menu, which contained foods with relatively high QI and DI scores, and the breakfast menu, which did not. Despite this difference, the combined meals had similar values for all three NBC parameters: DI = 0.55 & 0.46, QI = 1.21 & 1.44, NB = 83% & 81% for the lunch and breakfast, respectively.

**Fig 4:**
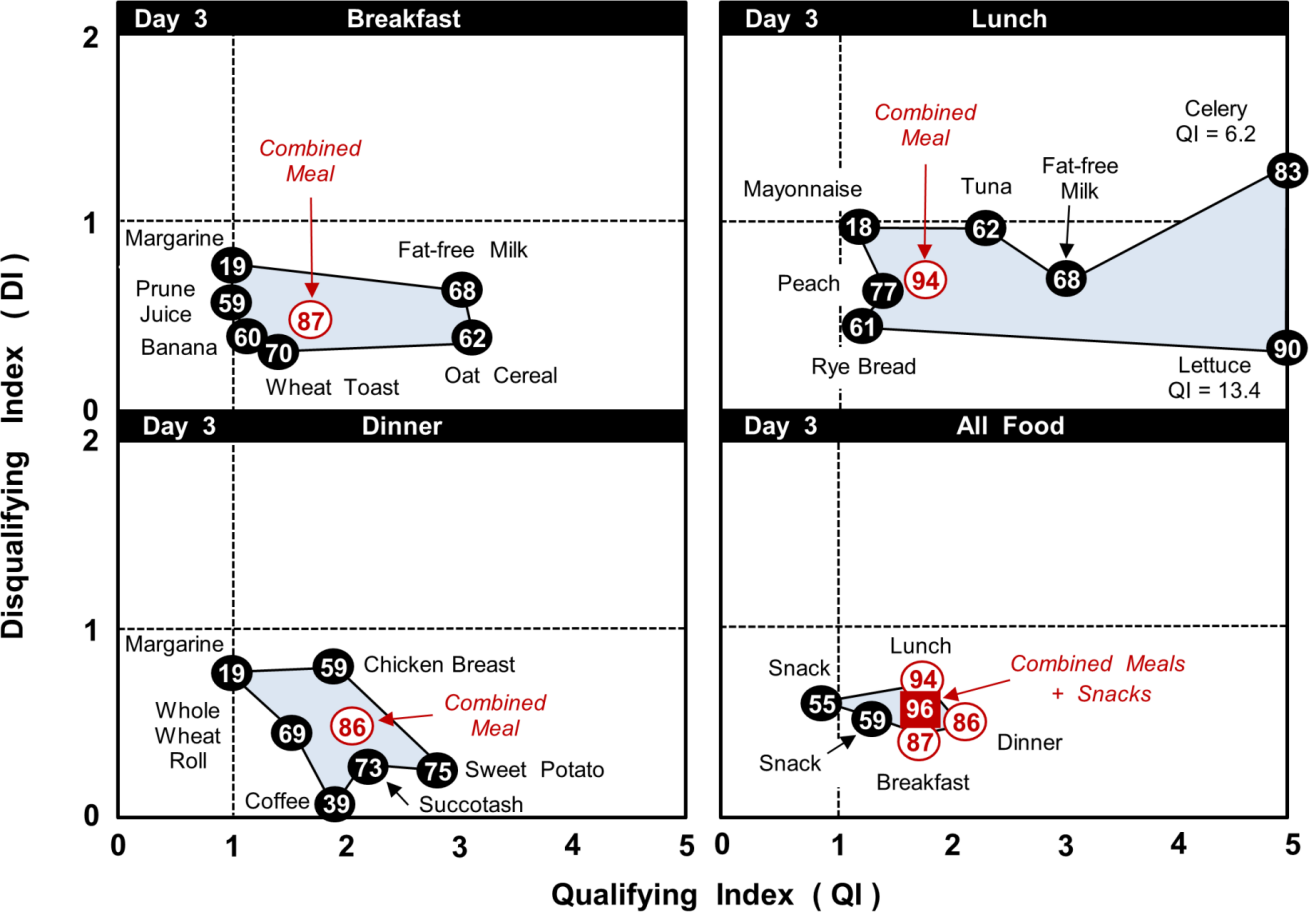
Day 3 (MyPlate 7-day menus): NBC Scores for foods, snacks and meals. Qualifying Index (QI), Disqualifying Index (DI) and % Nutrient Balance (NB, value in circle) for meal components, snacks, composite meals and the daily total food intake.

Also in contrast, Fig 4 shows that the meals on Day 3 were created from foods with much less variation in their individual values for DI, QI and NB than on Day 1. Again, the NBC parameters were very similar for the total food intake on both days: DI = 0.64 & 0.55, QI = 1.57 & 1.74, NB = 93% & 96% for Days 1 and 3, respectively.

Similar illustrations as Figs 3 and 4 but for Days 2, 4, 5, 6 and 7 of the MyPlate 7-day menus can be found in the ‘Supporting Information’ of this publication (respectively Figures A-E in S1 file).

A summary of the NBC parameters for each of the seven days and the total for the week is shown in Table 4. Again there was little variation in the values for DI, QI or NB between the individual days and the total for all 7 days (coefficient of variation < 11%).

**Table 4:** DI, QI and NB values for meals and snacks consumed on individual days and over the total 7-day period ^1^

Day	% of 7-day energy intake	DI	QI	NB
1	15.0	0.65	1.57	93.3
2	13.1	0.65	1.88	97.1
3	14.0	0.55	1.74	95.8
4	14.7	0.64	1.74	91.7
5	13.3	0.69	1.62	93.7
6	12.9	0.76	1.62	97.4
7	17.0	0.56	1.74	92.0
**1–7 Total**	**100.0**	**0.64**	**1.70**	**97.2**

1 ‘My Table’ Sample Menus for a 2000 calorie Food Pattern—Days 1–7 [20].

### Relationship between the Qualifying Index (QI) and the Nutrient Balance (NB) Scores

**Fig 5:**
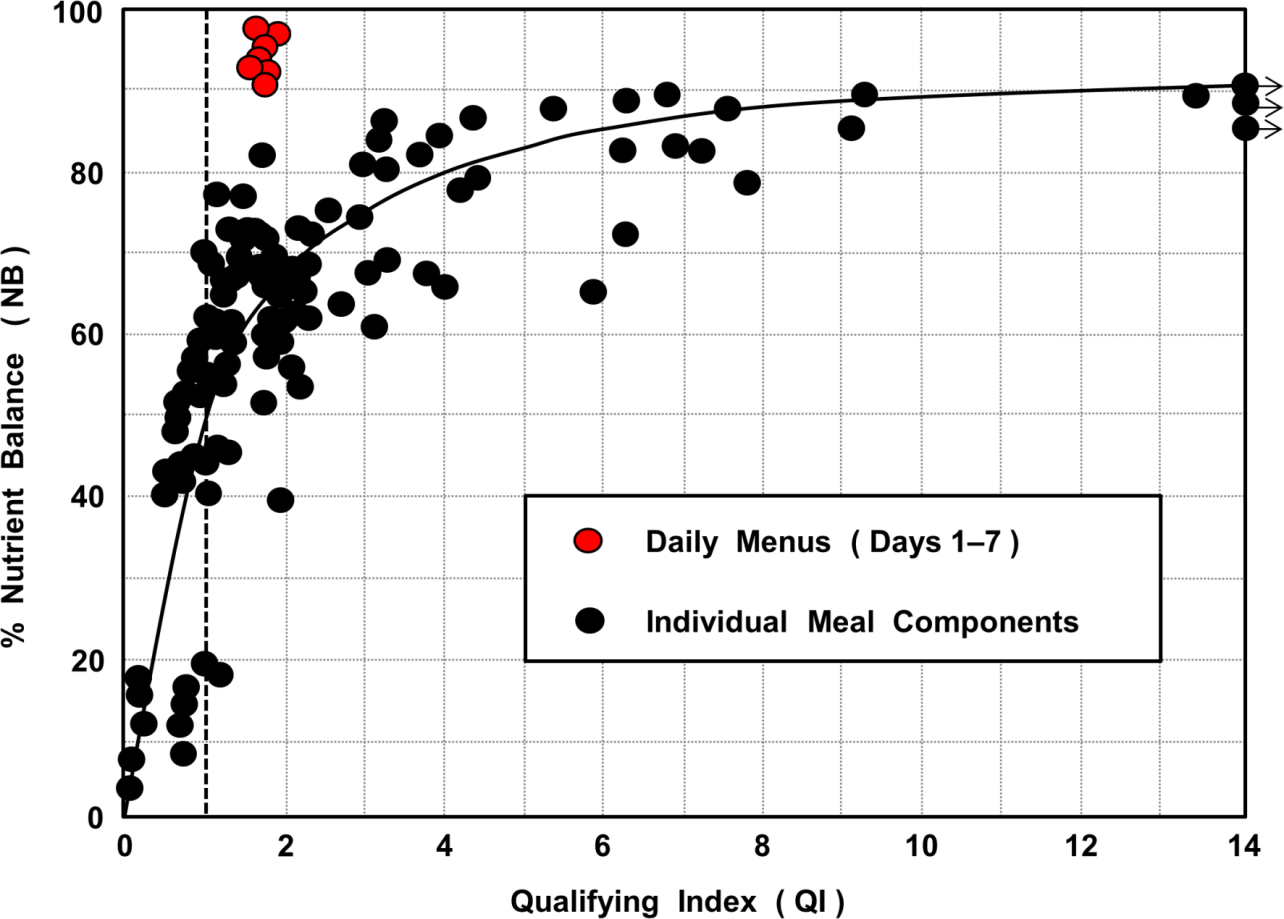
Relationship of Nutrient Balance to Qualifying Index (QI). Data shown are MyPlate 7-day meal components (black circles) and the combined meals and snacks for days 1 to 7 (red circles). The dotted line represents a ‘best fit’ curve for the meal components.

Fig 5 shows the direct relationship between QI and NB scores, based on the 132 principal food items used in the MyPlate 7-day menus. As would be expected, foods that were nutrient dense generally had higher NB values than did foods that were energy dense. However, for any given value of QI there was considerable variation in NB values, and *vice versa*. Some of the differences were as high as eight fold. Foods that had elevated QI and low NB scores were those that had unusually high levels of some qualifying nutrients but were relatively poor in many others.

When foods were combined in daily diets, however, and because of nutrient complementarity between foods, the relationship was markedly different to that for individual foods. Here much higher NB values (> 90%) were obtained at much low scores for QI (< 2.0).

### Complementarity and Non-complementarity of Foods

**Fig 6:**
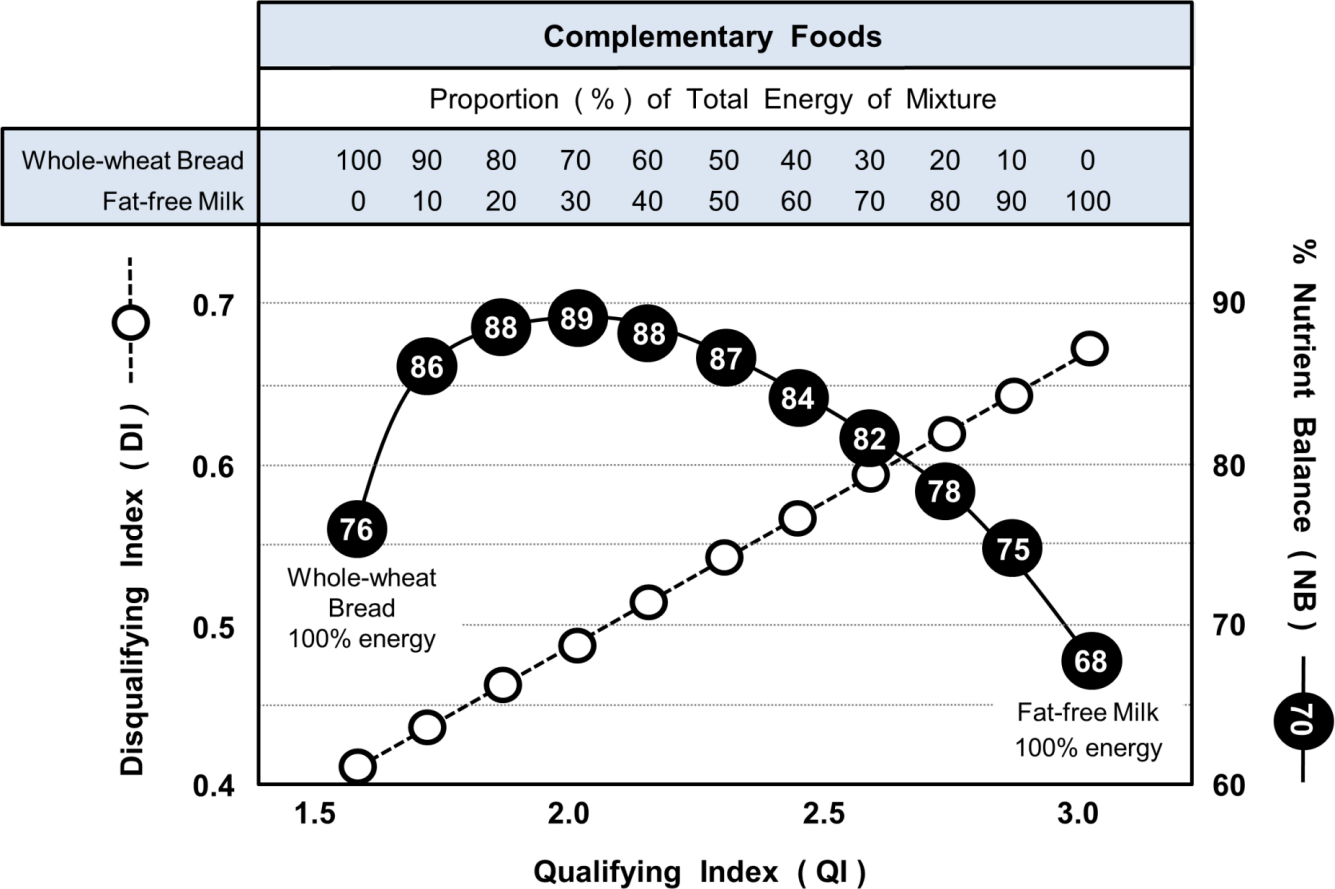
Complementarity shown by combining certain foods in varying proportions. Data shows the changing values of NB and DI relative to QI when whole-wheat bread and fat-free milk (Breakfast, Day 6, MyPlate 7-day menus) are combined in different proportions. Values in circles are % Nutrient Balance (NB).

**Fig 7:**
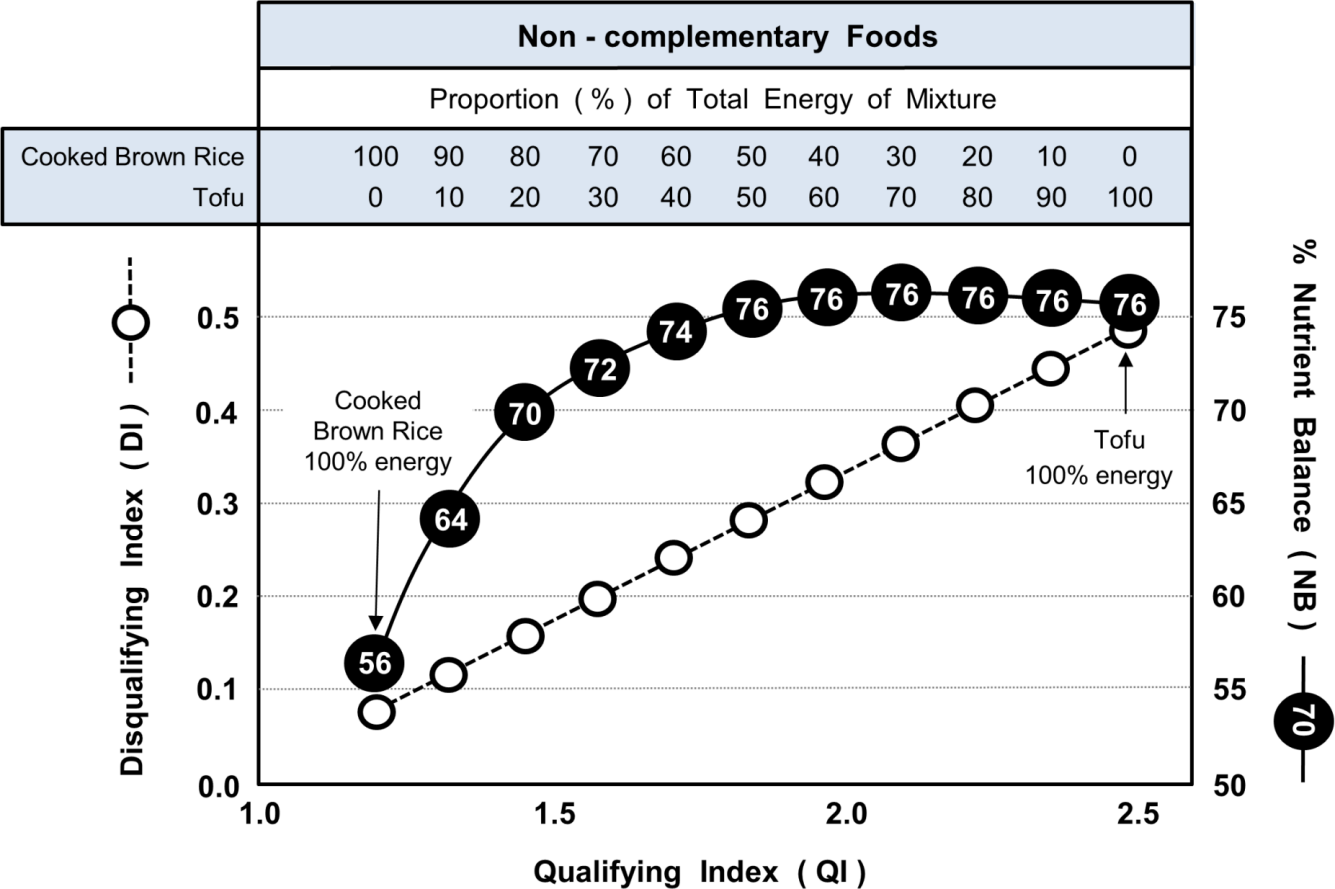
Non-Complementarity shown by combining certain foods in varying proportions. Data shows the changing values of NB and DI relative to QI when cooked brown rice and tofu (Dinner, Day 7, MyPlate 7-day menus) are combined in different proportions. Values in circles are % Nutrient Balance (NB).

Figs 6 and 7 illustrate, respectively, examples of complementarity and non-complementarity of foods in satisfying the dietary requirements of qualifying nutrients. This is analogous to the complementarity of amino acids in improving dietary protein quality. Shown are values of QI, DI and NB for a range of different combinations of the two major foods that provided over 50% of the total energy on Day 6 and 7 of the MyPlate 7-day menus. The combinations ranged from 100% of energy from one of the foods to 100% from the other.

Because QI and DI are both constants for a given food, the figures illustrate the predictable linear relationship between the Qualifying (QI) and Disqualifying (DI) Indices when energy contribution was varied. However, in sharp contrast, the relationship between the Qualifying Index (QI) and Nutrient Balance (NB) was very different for the two examples.

Fig 6 shows a high degree of complementarity between whole-wheat bread and fat-free milk (Breakfast, Day 6) as in all but two of the combinations shown, the Nutrient Balance (NB) exceeded that for the food with the highest NB score (whole-wheat bread). The maximum NB score (89%) was seen when whole-wheat bread provided 70% of the total energy and fat-free milk supplied 30%.

Fig 7 shows the opposite situation. The absence of any complementarity here is highlighted by the fact that in none of the combinations of cooked brown rice and tofu (Dinner, Day 7) did the NB value exceed that of the food with the highest NB score (tofu) and that half the tofu could be replaced with brown rice without changing the Nutrient Balance (NB) of the combination. Both aspects show that brown rice is unable to add net amounts of individual qualifying nutrients over and above those already provided by tofu.

## General Discussion

Nutrient profiling of individual foods has run counter to the long standing nutritional principle that there are no ‘good’ or ‘bad’ foods–only ‘good’ and ‘bad’ diets [24]. Indeed, many foods that are awarded low scores on standard nutrient profile models provide nutrients that are indispensable for maintaining health. Among such examples are monounstaurated and polyunsaturated fats in energy-dense nuts and calcium in whole milk. As studies have shown, nutrient-dense diets can be created using mixtures of both nutrient-dense and energy-dense foods [25]. Because most meals are composed of many different foods, there is a need to develop new metrics to evaluate nutrition quality, and correspondence between nutrients, of alternative food combinations in meals or total diets.

Past nutrient profiling models have made clear distinctions between energy-dense and nutrient-dense foods. Such models incorporated qualifying nutrients, mostly vitamins and minerals; and disqualifying nutrients, mainly fat, sugar, and sodium. Since fat and sugar are highly correlated with energy density, such nutrient profile models tended to award higher scores to foods that provided little dietary energy per unit volume. In other words, energy density and nutrient density of foods were inversely related.

The many technical challenges of developing nutrient profile models for foods and beverages have been described before [1–3]. These include the selection of qualifying and disqualifying nutrients (often described, respectively, as nutrients that encourage or limit health), the choice of reference daily values, and the base of calculation: 100g, 100 kcal, or serving size [1,26–29].

The Nutrient Balance Concept (NBC) helps to circumvent these important technical challenges and provides the first bridge between assessing the nutrient quality of individual foods and the overall nutrient quality of a combination of foods in meals or diets. Whereas variants of the Qualifying and Disqualifying Indices (QI and DI) have been used before, they have not been related to energy content in a systematic way and, therefore, to the numerical relationship between nutrient density and energy density. The Nutrient Balance (NB) component, which indicates the mean proportion of the daily requirements for multiple nutrients that are satisfied by a particular food or diet at the point when the daily energy requirements have been met, is an entirely new concept.

The scores for each of the three NBC parameters reflects a different aspect of nutrition quality and collectively they give a relatively complete description of it for any food, meal or diet. Their values that would describe an ‘optimum quality’ of a meal or diet are as follows:

- Disqualifying Index (DI) = less than 1.0

Values below 1.0 indicate that when daily energy intake is 2000 kcal, the average consumption of disqualifying nutrients will not exceed recommended Maximal Reference Values. Based on our experience to date, the mean DI score for meals and daily food intake is 0.8 + 0.3 (SD).

- Qualifying Index (QI) = 1.0

A QI value of 1.0 signifies that the average qualifying nutrient density is in proportion to that of energy for meeting daily dietary requirements. Other scores indicate the extent that the two densities differ. For example, a QI score of 2.0 would indicate the average qualifying nutrient density is twice that of energy, whereas one of 0.5 signifies that energy density is twice that of qualifying nutrients. However, because QI is an average value for the amount of qualifying nutrients present, it is a quantitative and not a qualitative measure of nutrient density. The qualitative aspect is provided by the Nutrient Balance (NB). The average QI score for composite meals and daily food intake that we have seen is 1.6 + 0.4 (SD).

- Nutrient Balance (NB) = 100%

A value of 100% for NB signifies that the dietary requirements for all qualifying nutrient considered are completely met at the point where energy needs are also satisfied (which in this study was taken to be 2,000 kcal).

Increasing NB values by simply raising the qualifying nutrient density (QI) of a food or diet, as for example by excessive fortification with vitamins and minerals, will effectively decrease the overall nutrition quality rather than improve it because fortification will disturb the equilibrium between the energy and qualifying nutrient density by automatically increasing the value of QI and moving it away from unity.

The average NB score we have observed is 59% + 22 (SD) for individual foods and food components, 87%+ 7 for composite meals, 92% + 3 for daily food intakes and 94% + 3 for 7-day intakes.

It is very unlikely, though, that in practice the three ‘ideal’ scores above will ever occur simultaneously in the same meal or diet. It then becomes a matter of conjecture as to which of the three parameters is the more important when comparing different foods, meals or diets. We would argue that all are equally important but that final decisions on choice of any particular recipe, menu or diet regime for a specific situation will also depend to a large extent on other important factors not covered in this publication. These include cost of ingredients, organoleptic aspects, cultural considerations and technical concerns, all of which can also be figured into the NBC algorithm if numerical data on these aspects is available.

One inherent limitation of the NBC is the requirement for an extensive nutrient composition database. The present NBC model was based on 27 qualifying nutrients–substantially more than are required by many current profiling methods. Previous studies have used as many as 23 different nutrients in different versions of nutrient density scores [1,2,27]. In contrast, others such as the SAIN (score for the nutritional adequacy of individual foods) method developed by Darmon et al for AFSSA, have been based on a very limited number of nutrients. The very limited selection in the SAIN study reflected the need to match nutrients of public health importance that were important markers of key food categories to markers for the presence of other nutrients, as well as the necessity to limit the nutrients to a manageable number [5].

The NBC approach can also be based, if required, on a smaller number of qualifying nutrients. Mathematical models based on 20 different foods covering the whole food spectrum and a total of 28 qualifying nutrients gave very comparable results when the number of qualifying nutrients were randomly decreased from 28 to 18 (results not shown). Past nutrient profiling models have been based on anywhere from 5 to 23 nutrients, most of which have provided essentially similar results.

Nevertheless, since there is a considerable and ever-increasing amount of data on food nutrient composition that is freely accessible today, it would be more reasonable and give better accuracy if as much data on nutrient composition as possible were used to judge quality of meals and diets.

The NBC is a versatile method that can easily be adapted to cover almost all dietary situations likely to be encountered in practice. Although, for example, the DRIs used in the current study have been those for women between 19–50 years of age (as a representation of the general population), the NBC can be tailored for all age groups (both male and female), for pregnancy and lactation, and for any other physiological condition for which dietary requirements are known.

It can also be adjusted for a specific nutrient of interest, either disqualifying or qualifying, for which more particular details are required. Thus, for example, if the effects of nutrient compositional changes on dietary sodium is of particular interest, the average content of all disqualifying nutrients on the ‘DI’ axis can be replaced with sodium values alone. Similarly, specific qualifying nutrients can replace the average value on the QI axis.

In addition, in the current study all nutrients have been considered to be equally important for health since the main objective was to show how the concept operated in practice. If it is considered that some nutrients require greater significance to be attached to their content in meals or diets, this can be realized simply through differential weighting of the di or qi values for the selected nutrients. The mathematical and statistical conditions of the concept, as described in the current study, still hold true for all the above adaptations.

Tolerable Upper Intake Level (UL)–the highest daily intake of a nutrient that is likely to pose no risks of toxicity for almost all individuals [30]–has not been considered in this study because it is extremely unlikely that in meals or diets with a QI value below 2.0, any of the individual nutrients will have reached their UL level, even with considerable overconsumption of food. Only if a multivitamin and mineral supplement supplying 100% of RDAs is taken regularly in addition to daily meals, will ULs require any consideration.

## Conclusions

The Nutrient Balance Concept (NBC) represents a new aspect to nutrient profiling. Although based on the nutrient composition of single foods, the NBC was specifically developed to take this a stage further–to be able to assess the overall nutritional quality of meals composed of multiple food items as well as that of total diets.

The three distinctive features of the method are novel metrics that:

1. Numerically describe the content of qualifying and disqualifying nutrients of individual foods in a way that is independent of portion size and which, importantly, acts as a solid basis for developing various algorithms
2. Allow the same analytical approach to be used across the entire food spectrum–from simple food components to complex meals and total diets, and
3. Provide an original way to judge nutrient complementarity and the overall nutrient quality of meals and diets composed of multiple food items.

The application of the NBC algorithm to the online MyPlate 7-Day Sample Menus provides the first objective evaluating of this important nutrition communication plan. The results showed that the overall nutrition quality of the meals developed by the USDA was very high in variety, total nutrient content and relative quantities of individual nutrients (see Figs 3, 4 and A-E in S1 File; Table 4). The nutrition quality of day-to-day food patterns was also very high and remarkably constant over 7 days. The NBC calculations did, nevertheless, show some variation in the values of QI, DI and NB across meals, depending on the specific foods used.

Finally, the NBC algorithm, which is relatively simple to create for particular applications and for different computer systems, could become a useful tool for devising diets or fine tuning meal planning, especially those for populations on a strict budget or with special cultural or clinical needs.

## Supporting Information

S1 File Qualifying Index (QI), Disqualifying Index (DI) and % Nutrient Balance (NB, value in circle) for meal components, snacks, composite meals and the daily total food intake. Contains Figures A-E. Figure A, Day 2 (MyPlate 7-day menus)—NBC Scores for Foods, Snack and Meals. Figure B, Day 4 (MyPlate 7-day menus)—NBC Scores for Foods, Snack and Meals. Figure C, Day 5 (MyPlate 7-day menus)—NBC Scores for Foods, Snack and Meals. Figure D, Day 6 (MyPlate 7-day menus)—NBC Scores for Foods, Snacks and Meals. Figure E, Day 7 (MyPlate 7-day menus)—NBC Scores for Foods, Snacks and Meals.
(ZIP)
