# Supplementary figures and images for: The Nutrient Balance Concept: A New Quality Metric for Composite Meals and Diets
Source: PLoS One. 2015 Jul 15;10(7):e0130491. doi: 10.1371/journal.pone.0130491 (PMC4503684; doi:10.1371/journal.pone.0130491)

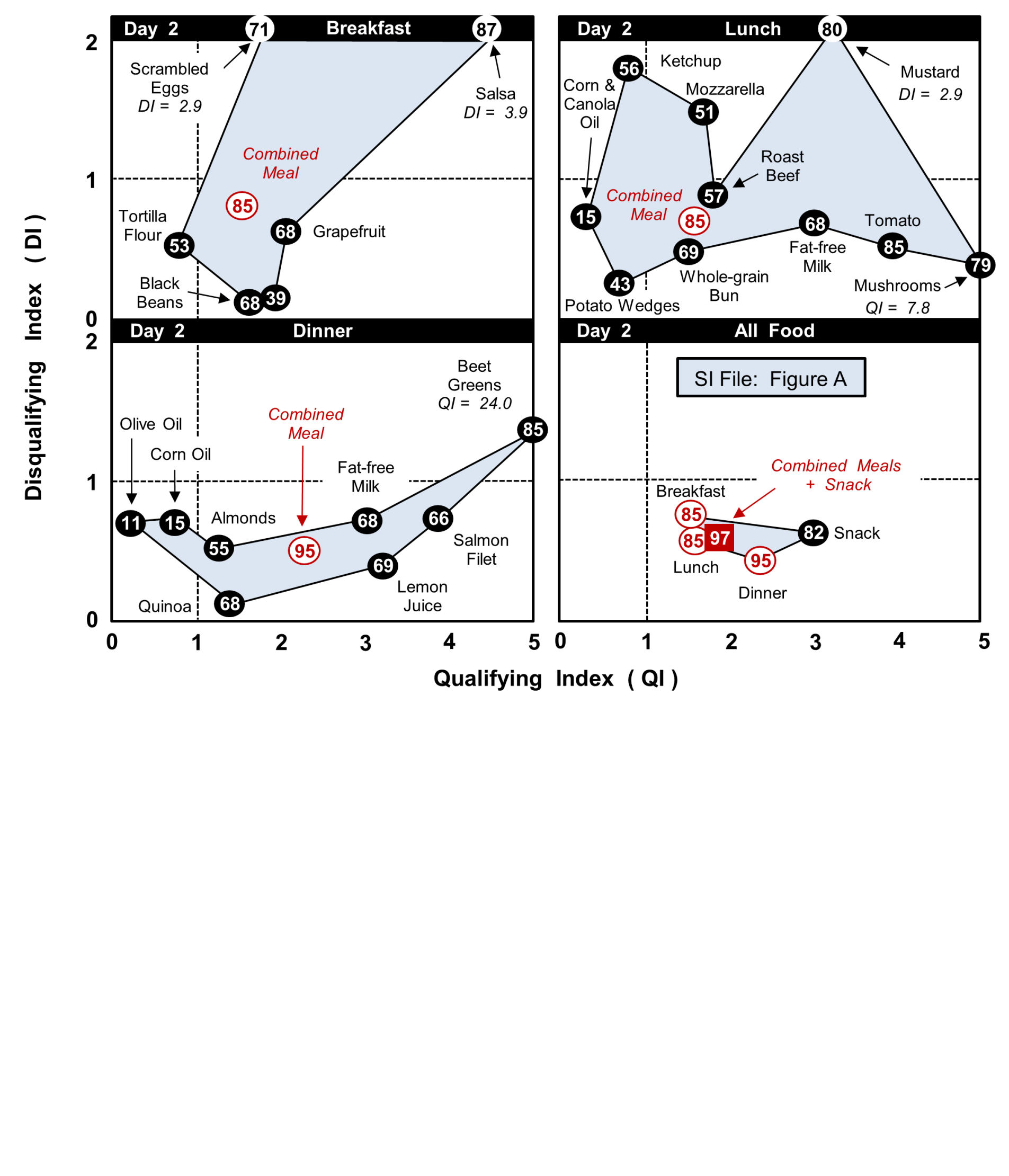

Supplement: S1 File — Contains Figures A-E. Figure A, Day 2 (MyPlate 7-day menus)—NBC Scores for Foods, Snack and Meals. Figure B, Day 4 (MyPlate 7-day menus)—NBC Scores for Foods, Snack and Meals. Figure C, Day 5 (MyPlate 7-day menus)—NBC Scores for Foods, Snack and Meals. Figure D, Day 6 (MyPlate 7-day menus)—NBC Scores for Foods, Snacks and Meals. Figure E, Day 7 (MyPlate 7-day menus)—NBC Scores for Foods, Snacks and Meals. (ZIP) [file pone.0130491.s001.zip › SI Figure A.TIF]

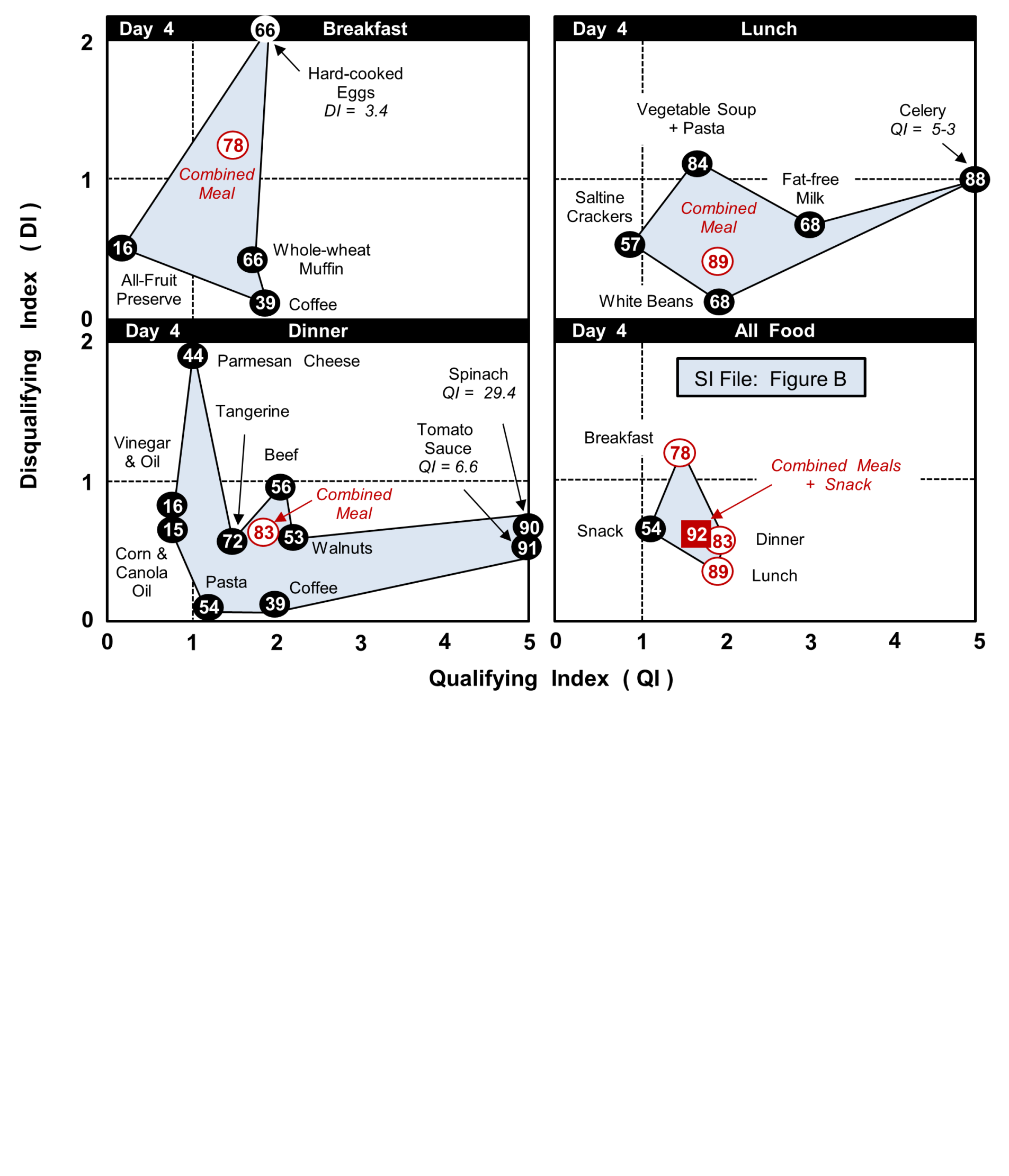

Supplement: S1 File — Contains Figures A-E. Figure A, Day 2 (MyPlate 7-day menus)—NBC Scores for Foods, Snack and Meals. Figure B, Day 4 (MyPlate 7-day menus)—NBC Scores for Foods, Snack and Meals. Figure C, Day 5 (MyPlate 7-day menus)—NBC Scores for Foods, Snack and Meals. Figure D, Day 6 (MyPlate 7-day menus)—NBC Scores for Foods, Snacks and Meals. Figure E, Day 7 (MyPlate 7-day menus)—NBC Scores for Foods, Snacks and Meals. (ZIP) [file pone.0130491.s001.zip › SI Figure B.TIF]

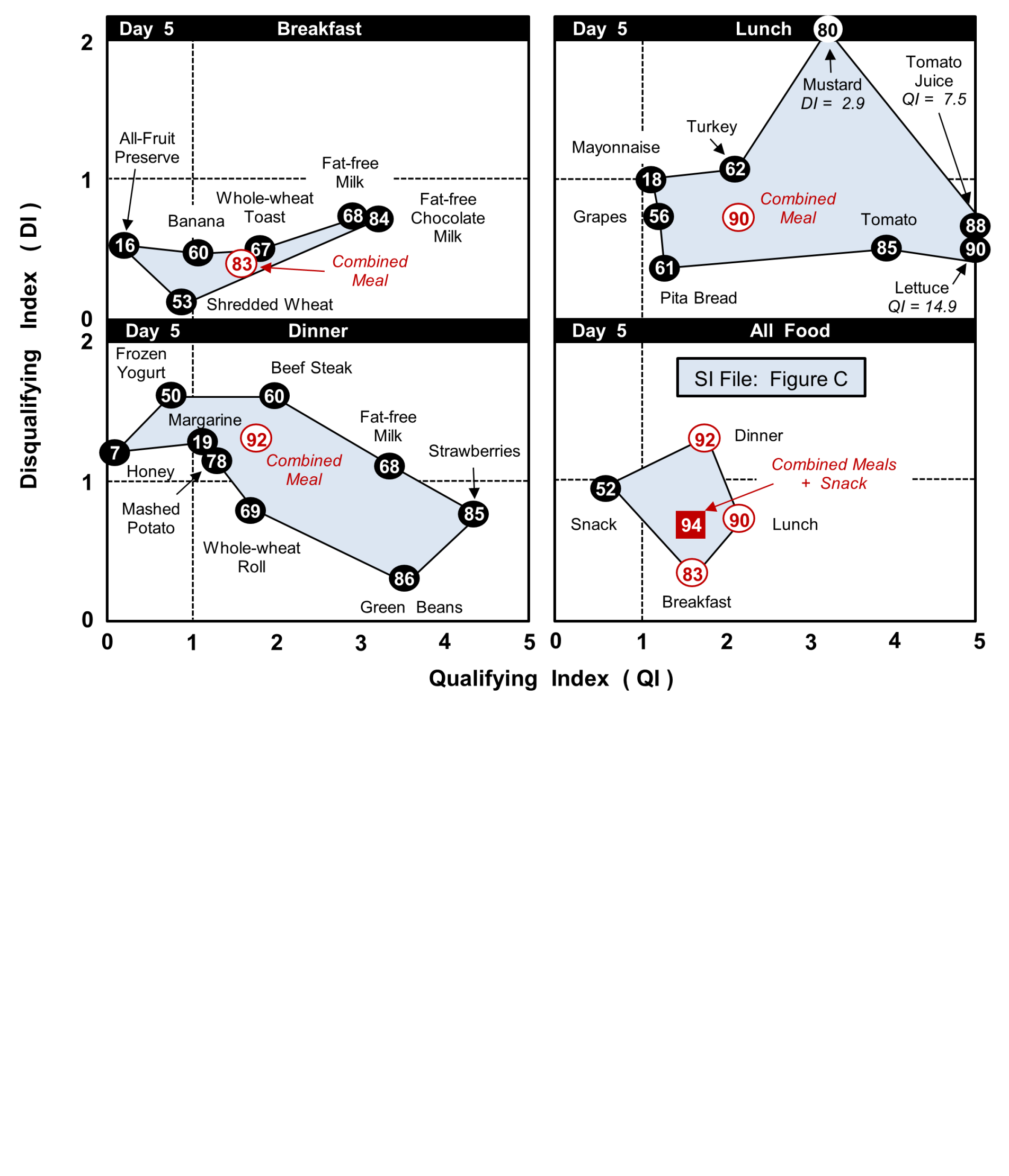

Supplement: S1 File — Contains Figures A-E. Figure A, Day 2 (MyPlate 7-day menus)—NBC Scores for Foods, Snack and Meals. Figure B, Day 4 (MyPlate 7-day menus)—NBC Scores for Foods, Snack and Meals. Figure C, Day 5 (MyPlate 7-day menus)—NBC Scores for Foods, Snack and Meals. Figure D, Day 6 (MyPlate 7-day menus)—NBC Scores for Foods, Snacks and Meals. Figure E, Day 7 (MyPlate 7-day menus)—NBC Scores for Foods, Snacks and Meals. (ZIP) [file pone.0130491.s001.zip › SI Figure C.TIF]

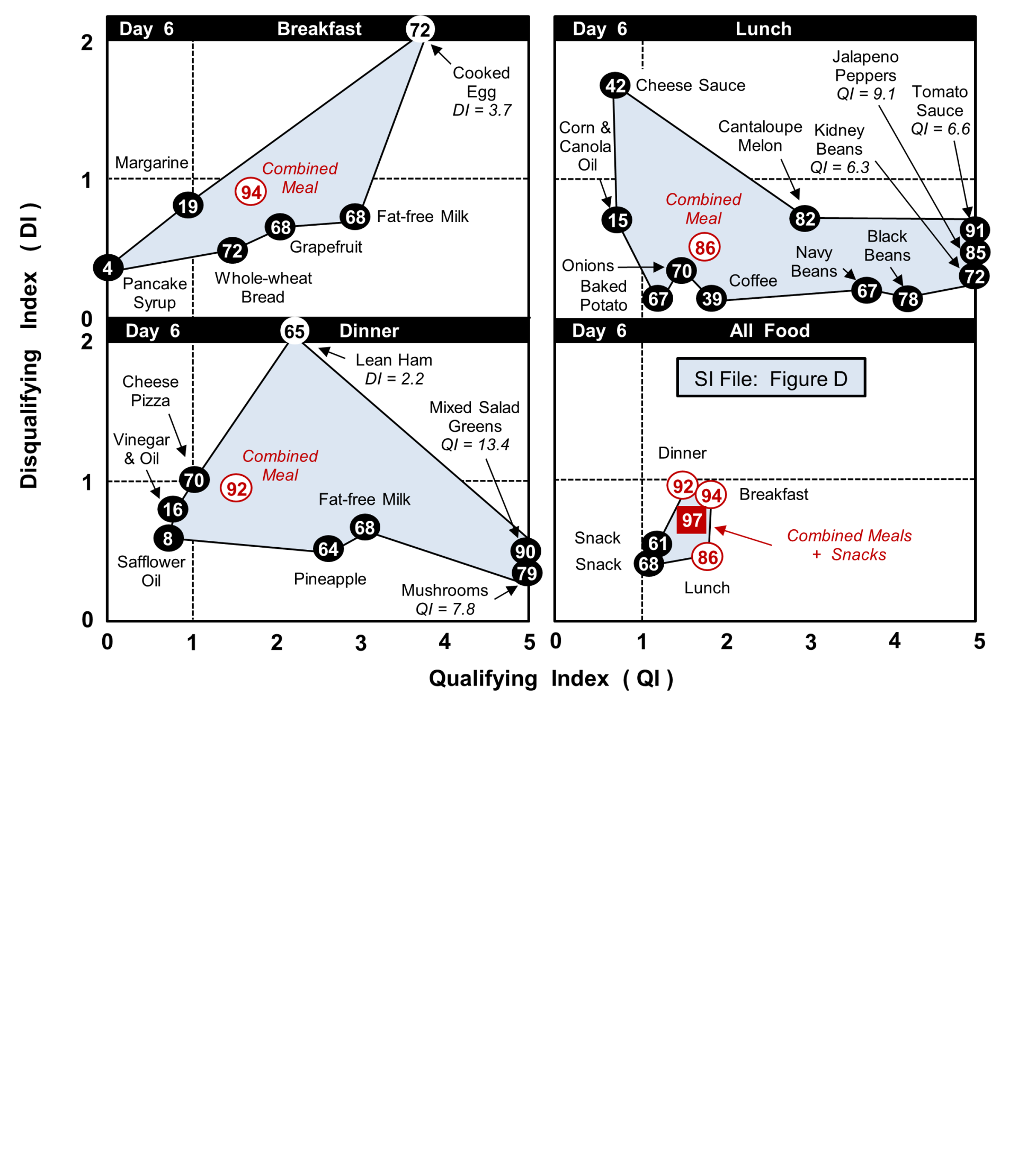

Supplement: S1 File — Contains Figures A-E. Figure A, Day 2 (MyPlate 7-day menus)—NBC Scores for Foods, Snack and Meals. Figure B, Day 4 (MyPlate 7-day menus)—NBC Scores for Foods, Snack and Meals. Figure C, Day 5 (MyPlate 7-day menus)—NBC Scores for Foods, Snack and Meals. Figure D, Day 6 (MyPlate 7-day menus)—NBC Scores for Foods, Snacks and Meals. Figure E, Day 7 (MyPlate 7-day menus)—NBC Scores for Foods, Snacks and Meals. (ZIP) [file pone.0130491.s001.zip › SI Figure D.TIF]

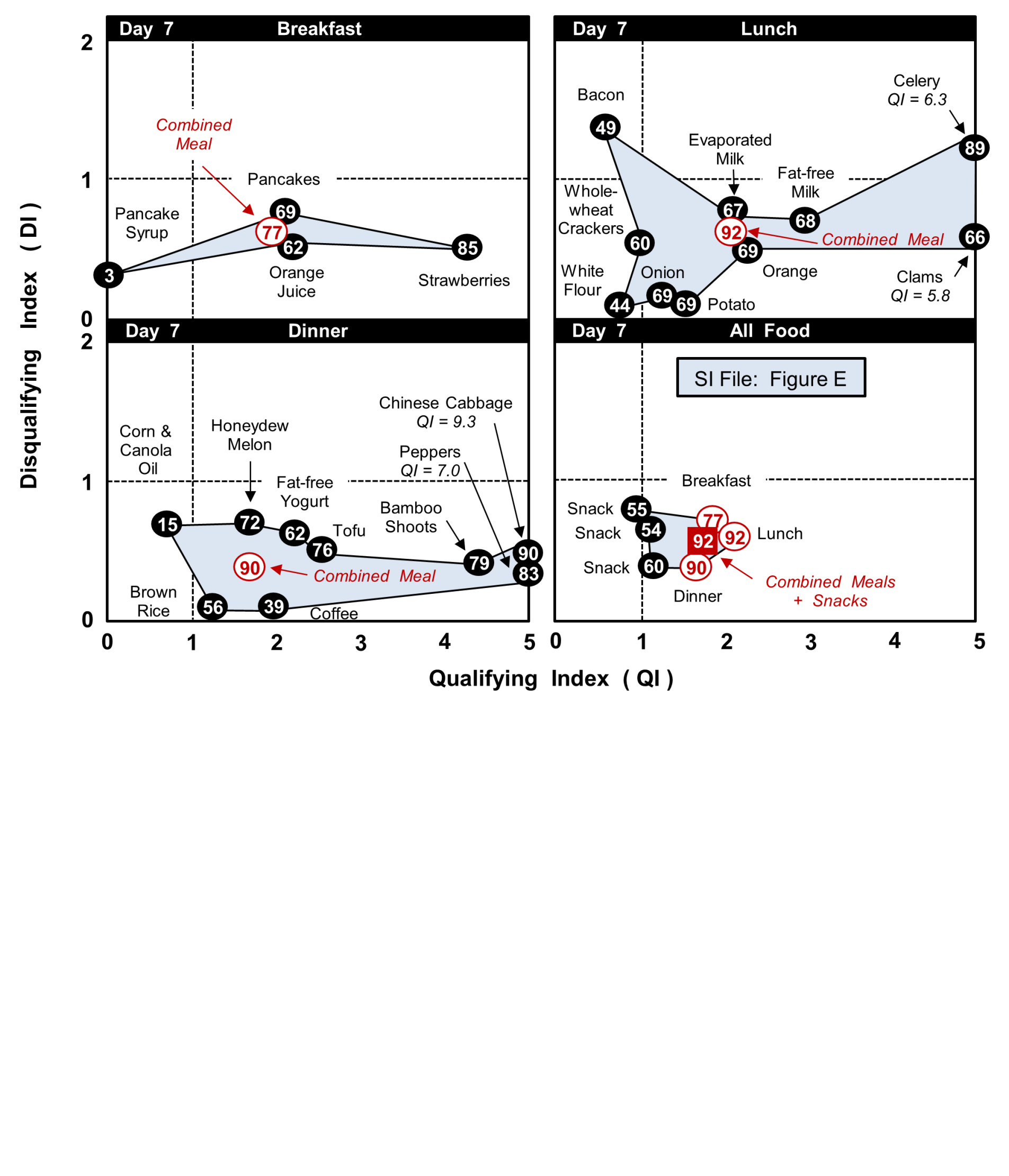

Supplement: S1 File — Contains Figures A-E. Figure A, Day 2 (MyPlate 7-day menus)—NBC Scores for Foods, Snack and Meals. Figure B, Day 4 (MyPlate 7-day menus)—NBC Scores for Foods, Snack and Meals. Figure C, Day 5 (MyPlate 7-day menus)—NBC Scores for Foods, Snack and Meals. Figure D, Day 6 (MyPlate 7-day menus)—NBC Scores for Foods, Snacks and Meals. Figure E, Day 7 (MyPlate 7-day menus)—NBC Scores for Foods, Snacks and Meals. (ZIP) [file pone.0130491.s001.zip › SI Figure E.TIF]
